# Study on the Evolution and Optimization of the Spatial Structure of the Oasis in the Arid Area: A Case Study of the Aksu River Basin in China

**DOI:** 10.3390/ijerph20064920

**Published:** 2023-03-10

**Authors:** Yunlu Jiang, Haotian He, Haoyu Zhang, Yuee Cao, Ge Shi, Lin Feng, Jianjun Yang

**Affiliations:** 1 College of Geography and Remote Sensing Sciences, Xinjiang University, Urumqi 830017, China; 2 Key Laboratory of Oasis Ecology, Xinjiang University, Urumqi 830017, China; 3 College of Life and Geographic Sciences, Kashi University, Kashi 844000, China; 4 College of Environmental and Geographical Sciences, Shanghai Normal University, Shanghai 200234, China; 5 School of Geography and Planning, Ningxia University, Yinchuan 750021, China; 6 School of Humanities and Law, Northeastern University, Shenyang 110169, China; 7 College of Ecology and Environment, Xinjiang University, Urumqi 830017, China

**Keywords:** land use, territorial spatial structure, suitability evaluation, territorial spatial optimization, Aksu River Basin

## Abstract

To achieve high-quality sustainable development in arid areas based on the concept of ecological civilization, it is necessary to deeply study the territorial spatial structure characteristics. Taking the Aksu River Basin, an important ecological security barrier in northwest China, as an example, this paper follows the research idea of “feature analysis-suitability evaluation-conflict identification analysis-optimization” and constructs a comprehensive model based on the AHP-entropy weight comprehensive evaluation method, ArcGIS spatial identification analysis, variance coefficient-TOPSIS method, and NRCA. A comprehensive model based on the AHP-entropy power integrated evaluation method, ArcGIS spatial identification analysis, variance coefficient-TOPSIS method, and NRCA was constructed to guide the optimization of the territorial spatial layout by exploring the characteristics of territorial spatial pattern, the suitability of territorial spatial development, the identification of territorial spatial conflicts, and the efficiency and functional advantage of territorial spatial utilization in the study area. The results show that: (1) The spatial type of territorial space in the Aksu River Basin from 2000 to 2020 is dominated by ecological space, agricultural space, and urban space, and the three spatial boundaries are irregularly interlaced. (2) The spatial utilization conflict pattern of the Aksu River Basin has formed, and the general conflict area is overgrowing. (3) The overall efficiency of territorial utilization in the Aksu River Basin is low, with significant differences among county administrative units. (4) After optimization, the three types of space in the watershed are adjusted and refined into six functional areas: basic farmland protection area, rural development area, ecological protection red line area, ecological control area, urban development area, and industrial supporting construction area.

## 1. Introduction

The unbalanced distribution of resources has become an influential factor in the sustainable development of nations around the world [1]. As the basic guarantee for human survival and development, the sustainable use of territorial space is an issue that has to be faced [2]. With the rapid growth of the population and continuous urbanization, problems such as resource shortage have intensified [3]. Most of the current studies on the territorial spatial structure are distributed in the spatial and temporal evolution characteristics of land use and land cover change (LULCC) and exploration of its driving factors [4,5,6], as well as on the dynamic changes of land use and its optimization [7] and the impact of land use changes on ecosystem services at different scales [8,9,10,11]. Territorial spatial structure is a comprehensive concept with rich connotations, generally referring to the reorganization of land use types used to meet the functional needs of ecological construction, agricultural construction and urban development, which covers ecological space, agricultural space and urban space [12,13]. Territorial spatial planning is a general plan for development and utilization management and layout optimization based on national resource conditions, economic and social development conditions, and strategies [14,15]. It embodies the research framework of “pattern-process-mechanism-diagnosis-optimization” in LULCC research [16]. Recent studies have focused on “carbon peaking and carbon neutrality”, climate change response, and regional coordination and sustainability. For example, Katarzyna et al. analyze the correlation between overall urban spatial layout changes and climate change in Poland [17]. Constance et al. analyze the benefits of integrated spatial planning through the lens of global demand for agricultural products [18]. Serraos et al. focus on regional adaptation actions in West Macedonia and assess their compatibility and complementary performance [19]. Geissler et al. conducted a sustainability planning study on NECP plans and land use types in Austria [20]. However, the relevant research is concentrated in the developed countries in Europe, and the research studies on arid areas are focused on separate sectors such as “arid areas meteorological climate change” [21], “arid areas water resources management” [22], “arid areas agricultural development” [23] and “arid areas desertification control” [24]. There is a lack of research on the overall coordination of territorial space, and hence, cross-administrative research still needs to be further strengthened. Land use control originated from the functional zoning system in Berlin after World War II [25]. The relevant theories and practices have been developed rapidly in recent years with different historical backgrounds and different emphases. Theoretical approaches include Kates’ multifunctional theory of territorial spatial zoning [26], Walter’s “central place theory” [27], Grant’s “cadastral theory model” [28], and Cerceau’s administrative-territorial theoretical framework [29]. With the development of computer technology, F. Wu and other scholars constructed the zoning theory based on geographic information technology and meta computer technology [30]. Generally speaking, there is an increasing trend toward quantitative analysis, but there have been many controversies about the quantitative criteria regarding economic and social factors [31]. From the perspective of research models, the integrated result–model optimization method has been widely used nowadays. It is primarily manifested in the introduction of “production-life-ecology” into spatial planning and the study of spatial delineation and optimization from the perspective of resource allocation [32], which has opened a new direction for the development of territorial space. However, the drawback of its excessive subjectivity also needs to be overcome [33]. Spatial optimization of territorial space in arid areas is a coordination involving many aspects such as national will, resource endowment, and people’s well-being, which requires strict formulation of optimization methods and paths [34,35]. It is worthwhile to think about how to use a combination of rigid constraints and flexible factors to eliminate spatial conflicts and achieve the optimization and reconstruction of the territorial spatial structure.

This paper selects the Aksu River Basin, an economic and social transition area with high ecological sensitivity, high population growth, and rapid urbanization, as the study area. To clarify the relationship between regional advantageous functions and land use changes, we have built a framework system of basin territorial spatial research coupled with “pattern-process-mechanism-diagnosis-optimization” to evaluate the characteristics of territorial spatial evolution, territorial spatial suitability, and conflict analysis from 2000 to 2020, to optimize the layout of territorial spatial structure in the Aksu River Basin. The research results provide a case study of the watershed perspective for the theory and method of territorial space optimization, and also provide ideas to solve similar problems for sustainable territorial space utilization in arid areas.

## 2. Materials and Methods

### 2.1. Study Area

The Aksu River is the largest water source among the three existing sources of the Tarim River (the largest inland river in China), accounting for more than 70% of the total water volume. The Aksu River Basin is located in the southwestern part of Xinjiang, China, the western part of the middle of the southern foot of the Tianshan Mountains, and the northwestern edge of the Tarim Basin [36]. The watershed area is about 57,000 km^2^. The climate is temperate continental arid climate [37].

**Figure 1:**
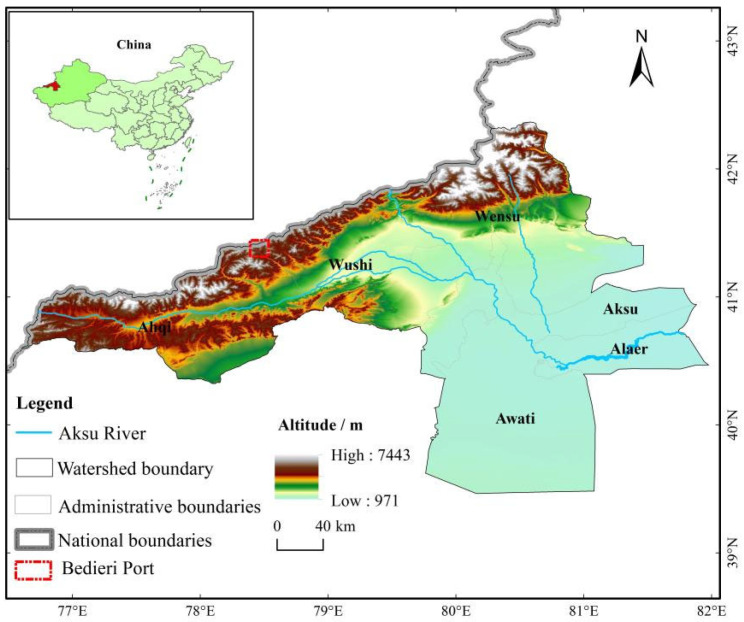
Summary map of the Aksu River Basin Study Area.

The Aksu River Basin area is developing unevenly. Moreover, it is also a significant area of ecological and environmental protection. With Xinjiang’s open ports’ construction to the outside world, the basin faces the double pressure of economic development and ecological protection [38]. The overall regional economic structure of the Aksu River Basin is an economic system with grain and cotton production as the mainstay and the orderly growth of light and heavy industries and services [39]. As of the end of 2019, the gross product of the Aksu River Basin was 73.77 billion RMB, with a household population of 1.55 million [40]. The per capita disposable income of urban and rural residents reached 32,800 RMB [41]. The study area is strategically located near the Kyrgyz Republic and the Republic of Kazakhstan, and the advantages of opening to the outside world, border trade, and border construction are obvious. The long-established and prestigious Beideri Port is located in Wushi county (as shown in Figure 1), and with the start of construction of the “China-Kyrgyzstan-Uzbekistan International Railway”, the status of the international logistics and commercial trade hub will be further strengthened [42].

The rapid advancement of urbanization and industrialization in the Aksu River Basin has prompted a fundamental transformation of the territorial spatial structure. Under such circumstances, the conflicts between human-land relations and unauthorized changes in land use attributes in the Aksu River Basin are becoming more and more serious. Therefore, carrying out the optimization of the land-space pattern is an important way to alleviate the conflict of land-space utilization in the Aksu River Basin and achieve high-quality development.

### 2.2. Data Resource

Land use data, meteorological data, traffic data, river and lake system data, population, and GDP, were obtained from the Data Center for Resource and Environmental Sciences of the Chinese Academy of Sciences. DEM data was obtained from the geospatial data cloud platform. Soil data was obtained from the China 1:1 million soil database of Nanjing Soil Institute, Chinese Academy of Sciences [43].

Relevant statistics were obtained from Xinjiang Statistical Yearbook 2000–2020, Aksu Regional Statistical Yearbook (2000–2020), Kizilsu Kirgiz Autonomous Prefecture Statistical Yearbook (2000–2020), China Urban and Rural Construction Statistical Yearbook 2000–2020, and China Rural Statistical Yearbook (2000–2020).

### 2.3. Research Procedure

**Figure 2:**
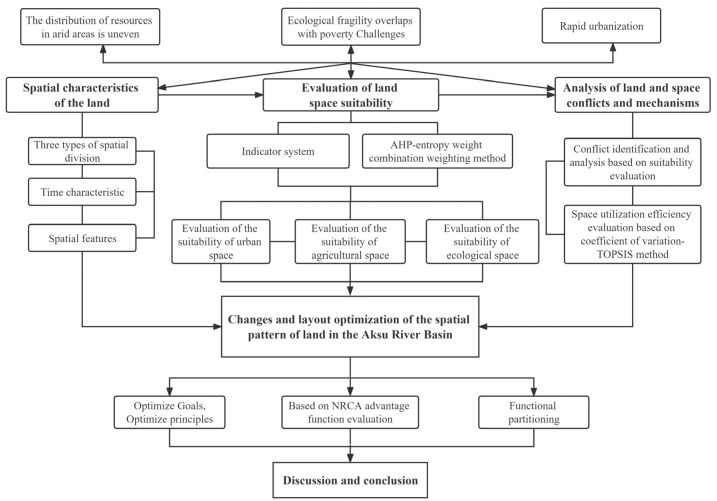
Technical roadmap.

The research procedure is shown in Figure 2.

### 2.4. Methodology

#### 2.4.1. Land Use Classification of Watershed Territorial Space

This paper, based on the interpretation results of Landsat 8 remote sensing data of the Aksu River Basin and the land use remote sensing monitoring classification data of the Chinese Academy of Sciences, classifies the study area into three categories of “urban space-agricultural space-ecological space” based on the concept of dominant territorial spatial function zoning as shown in Table 1 [44,45]. Such a classification principle is more conducive to the coordinated matching of land space functions and uses in arid areas watersheds with a complex topography and prominent spatial conflicts [46].

#### 2.4.2. Analysis of Land Use Dynamic Change

(1) Land use transfer matrix. The land use transfer matrix not only includes the area data of each category at a certain point of time in a certain region, but also has information of transferring out the area of each category at the beginning of the period and transferring in the area of each category at the end of the period, which reflects the dynamic process of mutual transformation between the area of each category at the beginning and the end of the period in a certain region at a certain time [47,48]. Its expression is:

(1) $$S_{\mathrm{ij}}=\left[\begin{array}{cccc} S_{11} & S_{12} & \cdots & S_{1n}\\ S_{21} & S_{22} & \cdots & S_{2n}\\ \vdots & \vdots & \vdots & \vdots \\ S_{n1} & S_{n2} & \cdots & S_{nn} \end{array}\right]$$

where *S_ij_* is the area of the initial *i* land use type transformed into the final period *j* land use type, and *n* is the total number of all land use types in the study area.

(2) Dynamics of single land use type. Its expression is as follows:

(2) $$K=\frac{U_{2}-U_{1}}{U_{1}}\times \frac{1}{T}\times 100\%$$

where *K*, *U*_1_, and *U*_2_ are the land use dynamics, period, and terminal land area, respectively; *T* is the study time period. The larger the absolute value of the dynamics, the faster the rate of land area increase or decrease [49].

(3) Comprehensive land use dynamics. Its expression is as follows:

(3) $$LC=\frac{\left(\sum_{i=1}^{n}\Delta LU_{i-j}\right)}{\left(2\sum_{i=1}^{n}LU_{i}\right)}\times \frac{1}{T}\times 100\%$$

where *LC*, Δ*LU_i_*_-*j*_, and *LU_i_* represent the annual rate of land use change, the absolute value of land use type *i* converted to non-*i* land use type, and the area of land use type *i* at the beginning of the study, respectively. *LU_j_* represents the area of a land use type at the end of the study. *T* is the year [50].

#### 2.4.3. Suitability Evaluation Method Based on AHP-Entropy Weight Method

(1) Analytic hierarchy process

Step one: Construct judgment matrix:

Evaluation indicators: *X* = {*X*1, *X*2, *X*3,……*Xi*}, among them, *i* = 1, 2, 3,……*n*.

Step two: Calculate the maximum eigenroot *λ*_max_ of the judgment matrix:

(4) $$\lambda_{\max}=\sum_{1}^{n}\frac{AW_{i}}{nW_{i}}$$

Step three: Calculate the consistency index ratio:

(5) $$CR=\frac{CI}{EI}$$

when *CR* < 0.1, it is generally considered to have passed the test. When the value is bigger than 0.1, the previously constructed judgment matrix needs to be adjusted until *CR* < 0.1 is met [51].

(2) Entropy weight method

In essence, the entropy weight method is an objective weight determination method. Its biggest advantage lies in the fact that human beings cannot intervene in the calculation process [52]. The steps of the evaluation index weight system constructed by the entropy weight method are as follows:

Step one: Standardization of data:

(6) $$X_{ij}=\frac{X_{ij}-X_{\min}}{X_{\max}-X_{\min}}$$

(7) $$X_{\mathrm{ij}}=\frac{X_{\max}-X_{\mathrm{ij}}}{X_{\max}-X_{\min}}$$

where *X_ij_* is the original value, *X*_max_ is the maximum value, and *X*_min_ is the minimum value.

Step two: Calculate the entropy value:

(8) $$E_{i}=-1/Ln*\sum_{i=1}^{m}a_{ij}Ln(a_{ij})$$

(9) $$a_{\mathrm{ij}}=X_{ij}^{\prime }/\sum_{i=1}^{m}X_{ij}$$

Step three: Calculate the difference coefficient, as shown in Formula (10):

(10) $$G_{i}=1-E_{i}$$

Step four: The weight value can be obtained, as shown in Formula (11):

(11) $$W_{i}=G_{i}/\sum_{i=1}^{m}G_{i}$$

Synthesis index method of weight:

(12) Xi=ziyi/m

(3) Indicator Evaluation System

According to the relevant technical guidelines issued by the Ministry of Natural Resources of China and based on the research of relevant scholars from the Chinese Academy of Sciences and Shanghai Normal University, this paper finally selected indicators suitable for the characteristics of the Aksu River Basin. The specific indicator system is shown in Table 2.

#### 2.4.4. Evaluation Method of Territorial Space Utilization Efficiency Based on the Coefficient of Variation-TOPSIS

(1) Coefficient of variation method

The coefficient of variation method is an objective indicator assignment method, which is based on the principle of taking the ratio of the standard deviation to the mean in the indicator system [53]. The greater the variation in the indicator’s value, the greater the weight share. The calculation process is as follows:

(13) $$d_{j}=\frac{\sigma_{j}}{{\bar{X}}_{j}}$$

(14) $$p_{2j}=\frac{d_{j}}{\sum_{j=1}^{n}d_{j}}$$

(2) TOPSIS method

The TOPSIS method is a method for conducting comprehensive evaluations and is suitable for multi-indicator situational calculations. The operation process ranks the closeness of evaluation objects according to their proximity to the desired goal, and determines the relative merits among the available objects [54,55]. The calculation process is as follows:

(15) $$W_{ij}=\frac{x_{ij}^{\prime }}{\sqrt{\sum_{i=1}^{m}x_{ij}^{\prime }{}^{2}}}$$

(16) $$\left\{\begin{array}{l} r_{j}{}^{+}=\max(r_{1J},r_{2j},\ldots ,r_{nj})\\ r_{j}{}^{-}=\min(r_{1J},r_{2j},\ldots ,r_{nj}) \end{array}\right\}$$

(17) $$\left\{\begin{array}{l} A_{i}{}^{+}=\sqrt{\sum_{j=1}^{n}{\left[b_{j}\left(r_{ij}-r_{j}{}^{+}\right)\right]}^{2}}\\ A_{i}{}^{-}=\sqrt{\sum_{j=1}^{n}{\left[b_{j}\left(r_{ij}-r_{j}{}^{-}\right)\right]}^{2}} \end{array}\right\}$$

(18) $$Ci=\frac{ri^{-}}{r_{i}{}^{{}_{+}}+r_{i}{}^{-}}$$

(3) Indicator Evaluation System

Following the current Guidelines for the Preparation of Municipal and County Territorial Spatial Master Plans (hereinafter referred to as the Guidelines) and the existing research results on the evaluation of the efficiency of the use of three types of spatial land, and based on the principles of high efficiency, quality, and quantification, the following index system was constructed by digging deeper into the essence of the three types of spatial land, while focusing on the principles of scientificity and comprehensiveness, dominance and accessibility in the selection of indicators. The details are shown in Table 3.

#### 2.4.5. Comparative Advantage Index

The NRCA (comparative advantage index) to classify the national space as a dominant function is expressed as.

(19) $$NRCA_{mn}=X_{mn}/X_{w}-X_{n}X_{mw}/(X_{w}X_{w})$$

where *X_mn_*, *X_mw_* denote the area of type *n* space of county m in the study area and the total area of county country land, respectively. *X_n_*, *X_w_* denote the area of type *n* space in the study area and the total area of country land space in the study area, respectively. The dominance index > 0 indicates that the n type of country land in county m has comparative advantage. The dominance index < 0 indicates that the n type of country land in county m has no comparative advantage [56]. The occurrence of two types > 0 at the same time is classified as a single type if the difference is large.

#### 2.4.6. Optimization Methods

**Figure 3:**
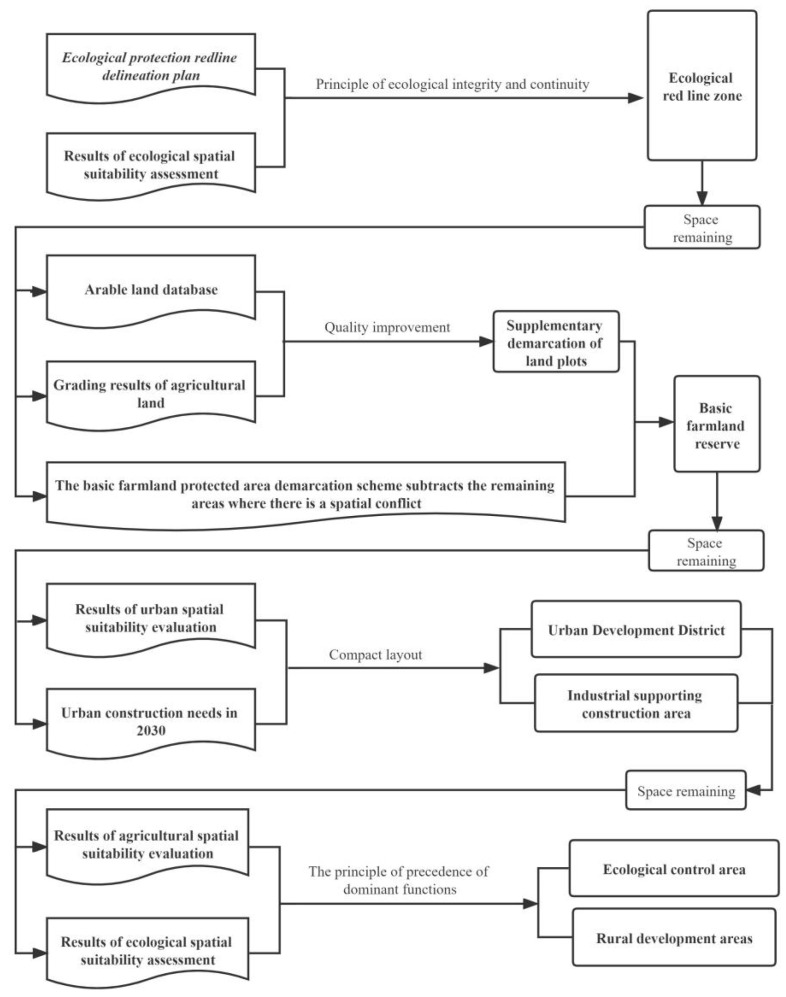
Map of the process of optimizing the territorial spatial structure layout.

The optimization of the territorial spatial layout should give priority to guaranteeing the sustainable development of territorial space, focusing on the delineation of various rigid protection zones that guarantee the ecological security of territorial space and the safety of agricultural production. The needs of urban construction, urban development, and construction areas should also be delineated. Finally, the remaining spatial functional areas should be determined according to the suitability level and regional dominant functions (Figure 3).

## 3. Results and Analysis

### 3.1. Analysis of the Spatial and Temporal Evolution of the Three Types of Spatial Patterns

**Figure 4:**
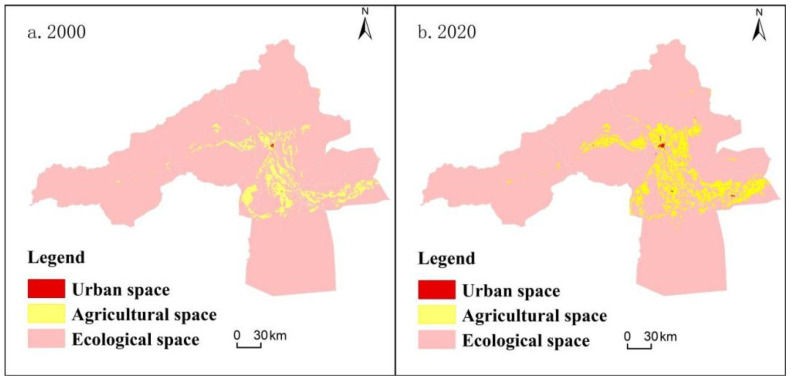
Land spatial distribution map of Aksu River Basin in 2000 and 2020.

As shown in Table 4, the spatial pattern of the study area is basically compatible with the regional physical geography and socio-economic operation. Ecological space is dominant in the Aksu River Basin, followed by agricultural and then urban spaces. From 2000 to 2020, the three types of spatial changes in the Aksu River Basin are characterized by “two increases and one decrease”. The urban space increased by 304.67%, the agricultural space increased by 49.01%, and the ecological space decreased by 5.68%. The expansion of urban and agricultural space is apparent, reflecting the rapid development of urbanization and agricultural land in the Aksu River Basin during the study period, the details are shown in Figure 4 and Table 5. The agricultural space in the study area is mainly distributed in the valley and alluvial fan edge of the Aksu River Basin and the middle and lower reaches of the irrigation area. The administrative division is mainly distributed between the middle and downstream parts of Aksu city, Awati county, Wensu county, and Alaer city. The urban space is mainly distributed in areas with abundant freshwater resources and convenient transportation. A town pattern has formed with administrative center sites at all levels as the center and major transportation routes as the spreading wings. The ecological space is mainly laid out in the middle and upper mountainous regions and the Taklamakan Desert. It is mainly distributed in Aqki and Awati counties according to administrative divisions.

### 3.2. Suitability Evaluation Results

Based on the Arcgis 10.7 platform, the spatial suitability of the Aksu River Basin was classified into four categories: most suitable, suitable, less suitable, and unsuitable areas, mainly using the natural discontinuity method. Among them, the most suitable, suitable, less suitable and unsuitable areas for urban space were 7064.41 km^2^, 15,499.63 km^2^, 21,723.96 km^2^, and 12,433.88 km^2^, respectively. The proportions of each type were 12.45%, 27.33%, 38.30%, and 21.92%, respectively (Table 6). The most suitable, suitable, less suitable, and unsuitable areas for agricultural space were 955.14 km^2^, 2515.39 km^2^, 939.97 km^2^, and 286.57 km^2^, respectively. The proportions of each type of area were 20.33%, 53.55%, 20.01%, and 6.10% (Table 7). The ecological spatial optimum, suitable, less suitable, and unsuitable areas were 5984.17 km^2^, 16,221.52 km^2^, 13,090.16 km^2^, and 21,696.94 km^2^, respectively. The proportion of these areas were 10.50%, 28.46%, 22.97%, and 38.07%, respectively (Table 8).

**Figure 5:**
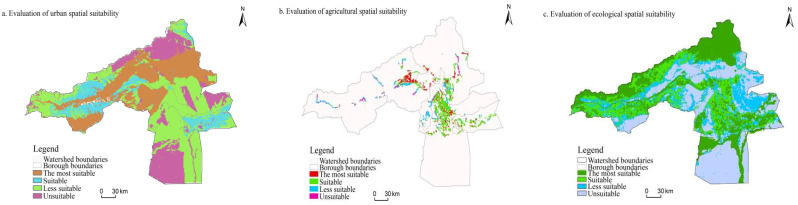
Map of the results of the evaluation of the suitability of land space.

According to the spatial structure (Figure 5), the most suitable areas and suitable areas for urban space are mainly located in the middle and lower reaches of river valleys and alluvial plains in the watershed with sufficient water supply, flat water supply terrain, convenient transportation, and less geological hazards. By administrative district, these areas are mainly located in Aksu city. The most suitable and suitable areas for agricultural production are mainly distributed in the middle, and lower reaches of the Aksu River irrigated agricultural area, accounting for more than 60% of the whole basin. By administrative districts, they are mainly distributed in Awati county and Wensu county. The most suitable and suitable areas for ecological space are mainly in river valleys, high-altitude glacier areas, and oasis edges; Ahechi, Wushi, and Wensu counties have a high area share because of the large number of multi-alpine areas and glaciers distributed in their territories. The more unsuitable and unsuitable areas in urban space are mainly located in Ahechi county, Wensu county and Awati county. Awati county is because most of the area is a desert region with harsh natural conditions. The undulating terrain in their territories causes problems in Wensu and Ahechi counties. The unsuitable areas for agricultural space are mainly in high mountains, river valley areas and from the edge of the oasis, and some urban built-up areas. Many mountainous areas in Wensu, Ahechi, and Wushi counties make regional agricultural development difficult. Awati county has the highest percentage of unsuitable ecological areas, as most of its territory is desert with little surface vegetation cover and minimal natural resources.

### 3.3. Spatial Conflict Identification

#### 3.3.1. Territorial Space Contradiction Conflict Distribution

**Figure 6:**
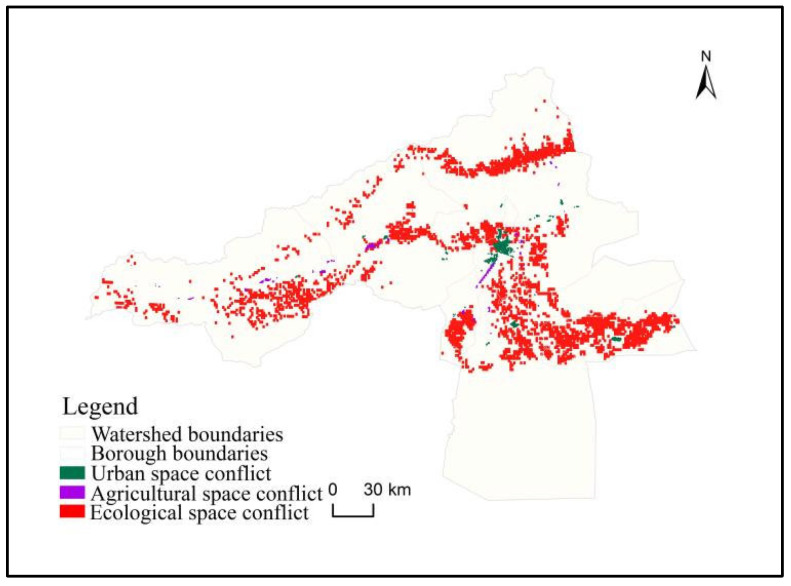
Distribution diagram of territorial spatial conflicts in the study area from 2000 to 2020.

The spatial conflict pattern of the country in the Aksu River Basin from 2000 to 2020 is examined in Figure 6. The conflict zone has prominent pattern characteristics of overall dispersion and detailed agglomeration. From the spatial conflict of urban space, the conflict zones are mainly concentrated in the south-central floodplains of the Aksu River basin. There are many conflict areas regarding agricultural spatial conflicts, but each is very small. From the perspective of ecological spatial conflicts, they are the most widely distributed in the area and are closely related to the dense human activity areas.

#### 3.3.2. County Land Spatial Pattern Conflict

**Figure 7:**
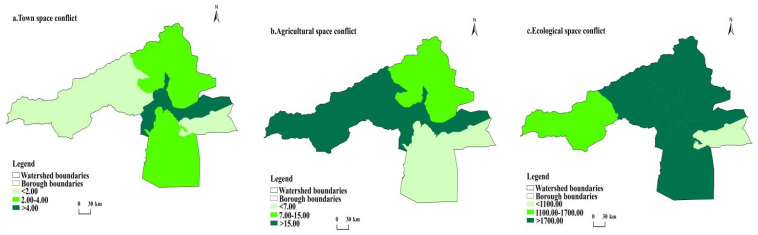
Evaluation map of territorial spatial conflict in 2000.

**Figure 8:**
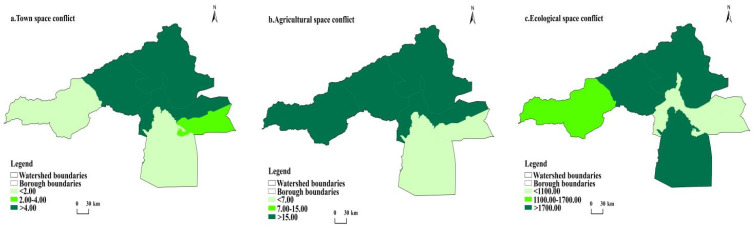
Evaluation map of territorial spatial conflict in 2020.

In this study, the spatial layout of the conflict situation is divided into three levels according to the natural interval method. (Figure 7 and Figure 8).

From the spatial conflict of urban space, low conflict (<2 km^2^) counties in 2000 were Ahqi and Wushi counties and Alaer city. In 2020, they were Awati and Ahqi counties. The medium conflict (2–4 km^2^) counties were 2 in 2000, namely, Wensu county and Awati county. The medium conflict area in 2020 was Arar city. High conflict (>4 km^2^) counties were Aksu city in 2000, and Wushi county, Wensu county and Aksu city in 2020.

Regarding agricultural spatial conflicts, low-conflict counties (<7 km^2^) were Alaer city and Awati county in 2000 and 2020. The medium-conflict counties (7–15 km^2^) were Wensu county in 2000. The high-conflict counties (>15 km^2^) were Aksu city, Wushi county, and Ahqi county in 2000, and Aksu city, Wushi county, Wensu county, and Ahqi county in 2020.

From the perspective of ecological spatial conflicts, low-conflict counties (<1100 km^2^) were Alaer city in 2000, and Aksu city and Alaer city in 2020. The medium conflict county was (1100–1700 km^2^) Ahqi county. The high-conflict counties (>1700 km^2^) were Ahqi county, Awati county, Wushi county, and four counties of Aksu city in 2000. In 2020 they were Wensu county, Wushi county, and Awati county.

Generally, there are significant differences in the spatial conflicts of county towns, agricultural spatial conflicts, and ecological spatial conflicts in the Aksu River Basin from 2000 to 2020. However, in terms of overall spatial distribution, the conflict areas are primarily concentrated and have strong spatial and temporal correlations. The irrational development situation has eased in momentum in recent years, but policies still influence it greatly, and the possible range of future variables is still very large. We can only say that risks still exist, but we can retain a more optimistic expectation for the future.

### 3.4. Spatial Utilization Efficiency of the Land

**Figure 9:**
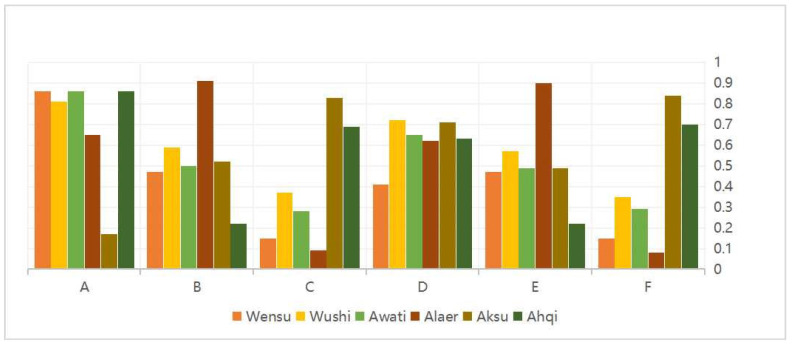
Map of the evaluation of the efficiency of land space utilization. A. Spatial efficiency index of urban use in 2000. B. Spatial efficiency index of agricultural use in 2000. C. Spatial efficiency index of ecological use in 2000. D. Spatial efficiency index of urban use in 2020. E. Spatial efficiency index of agricultural use in 2020. F. Spatial efficiency index of ecological use in 2020.

The land use efficiency indicators of urban, agricultural, and ecological space in the study area were calculated based on the coefficient of variation-TOPSIS method, details are shown in Figure 9. The utilization efficiency of urban space in the Aksu River Basin is generally high. In 2000, the utilization efficiency of all counties and cities was above 80%, except for Alaer city and Ahqi county, which were low. This indicates that the built-up area of towns and cities in 2000 and before was small, the vitality of outreach and expansion was insufficient, and the level of regional economic development needed to be improved. The reason for the low efficiency of Alaer city was the short time since its establishment and the vague planning and positioning. The large decrease in utilization efficiency of Wensu county and Aksu city in 2020 compared to 2000 is due to the rapid economic development of the two counties and cities, which are the locations of major regional administrative agencies. There was a significant increase in the area of urban construction land and industrial and mining land (mainly logistics centers, industrial estates, economic development districts, and oil fields), but the population growth rate has not kept pace with the growth rate of construction land, thus leading to a significant decrease in utilization efficiency. Regarding individual indicators, the average value added of secondary and tertiary industries in the Aksu River Basin is the highest in Alaer, which indicates that Alaer has a good foundation for industrial development and conditions for service industry development.

The efficiency of agricultural spatial land use in the Aksu River Basin, in general, has clear differences among counties and cities. The higher the efficiency of agricultural space utilization, the higher the proportion of income from primary industry among its three industries. The slower the industrialization process, the worse the overall economic development level. Ahqi county has dominated by primary industries and has great difficulties in transformation because of its geographical location and resource endowment limitations. The large change in Alaer city within ten years indicates the rapid socio-economic development and decisive adjustment of industrial structure layout in the last ten years.

The overall low efficiency of ecological space land use in the Aksu River Basin is due to many difficult-to-use areas such as desert and alpine glaciers. The highest utilization efficiency is in Alaer city, and the lowest is in Ahqi county. The poor allocation of resources in Ahqi county, coupled with the construction of large-scale agricultural construction and urban facilities, has led to a decrease in forest and grass cover and a reduction in the area of ecological space land. Alaer city is located in the middle and upper reaches of the Tarim River, with sufficient water resources, coupled with better topography and heat conditions, resulting in a wide area of forest and grass in the ecological space, and the area of artificial forestation is growing fast, keeping the overall utilization efficiency high.

### 3.5. Territorial Spatial Functional Advantage of Aksu River Basin

**Figure 10:**
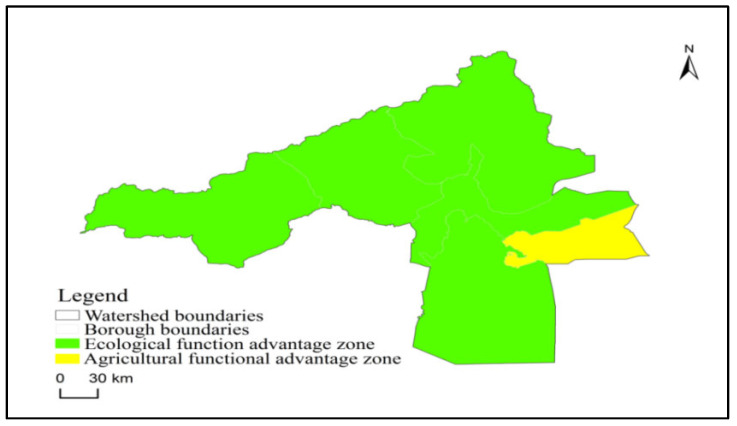
Spatial distribution of advantage functions in the Aksu River Basin.

From the distribution pattern of the comparative advantage function in the Aksu River Basin (Table 9 and Figure 10), the comparative advantage pattern of the “Town-Agriculture-Ecological space” is quite different. However, the trend of group development is noticeable. As a whole, the basin is dominated by the ecological advantage function. This is due to the vast area of unused land in the basin, which is over-represented. By subdivision, the areas with more visible advantages of urban functions are mainly located in Aksu city, Wensu county, and Alaer city in the middle and lower reaches of the river plain and the southeastern part of the basin. The areas with more obvious advantages in agricultural functions are mainly the vast plain irrigation areas in the basin’s hinterland and the gently sloping valleys of Alaer city, Aksu city, Wensu county, and Awati county. The areas with obvious eco-spatial functional advantages are mainly Ahqi county, Wushi county, and Awati county, which are located in the alpine snow cover area and the belly of Taklamakan Desert. The classification of Aksu city as an eco-spatially advantageous area is caused by its high values of key indicators such as vegetation cover and coordination of soil and water resources.

### 3.6. Results of Territorial Spatial Optimization in the Aksu River Basin

Optimizing the spatial layout of the territorial space should eliminate the contradictory conflicts in the territorial space and improve the efficiency of urban space utilization. Firstly, various types of rigid protection space should be delineated to guarantee regional spatial security. On this basis, urban space should be delineated to meet the reasonable construction needs of towns and cities, and the space beyond that should be delineated as general agricultural space and general ecological space according to the principles of high suitability grade priority and dominant function priority. After optimization, the territorial space will be divided into three major spaces and six functional areas, which are ecological space containing ecological protection red line areas and ecological control areas, agricultural space containing basic farmland protection areas and rural development areas, and urban space containing urban development areas and industrial supporting construction areas.

**Figure 11:**
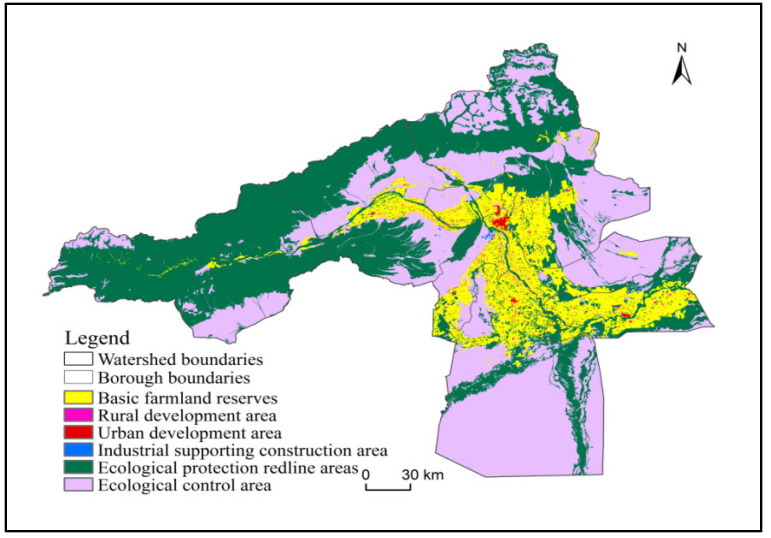
Aksu River Basin Territorial Space Optimization Zoning Map in 2030.

**Figure 12:**
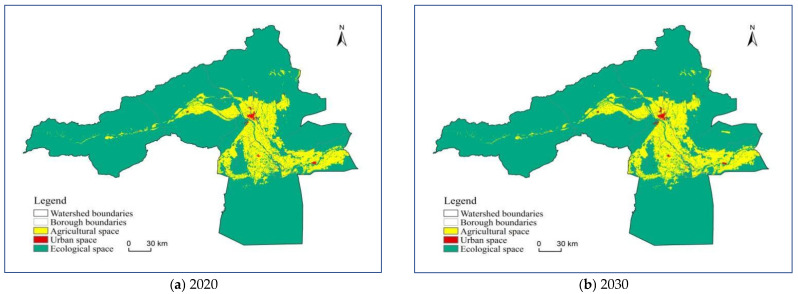
Territorial and spatial distribution map of the Aksu River Basin in 2020 and 2030.

The data is firstly analyzed and visualized by layer data extraction based on Arcgis 10.7 platform, and the boundary is determined according to the regional territorial spatial function dominance by identifying various secondary land class attributes. The new spatial structure classification boundary of the land is based on the Chinese land use remote sensing monitoring dataset provided by the Chinese Academy of Sciences. This dataset is based on the Chinese National Resources and Environment Database and uses the Landsat series remote sensing image data from the U.S. Landsat as the main information source, with high and authoritative data accuracy. According to the regional development planning needs in 2030, the optimization results are obtained through definite quantitative analysis. According to the optimization results (Table 10 and Table 11, Figure 11 and Figure 12), the areas of ecological, agricultural and urban spaces in Aksu River Basin are 48,729.98 km^2^, 8793.87 km^2^ and 157.61 km^2^, respectively, which account for 84.48%, 15.25% and 0.27% of the territorial space in Aksu River Basin, respectively. That is, in 2030, the territorial space of Aksu River Basin will still be ecological space > agricultural space > urban space, which is in line with its main function positioning as the ecological barrier on the southern slope of Tianshan Mountain. In terms of change rate, the growth rate of agricultural space is higher than urban and ecological space.

## 4. Discussion and Implications

### 4.1. Discussion

**Figure 13:**
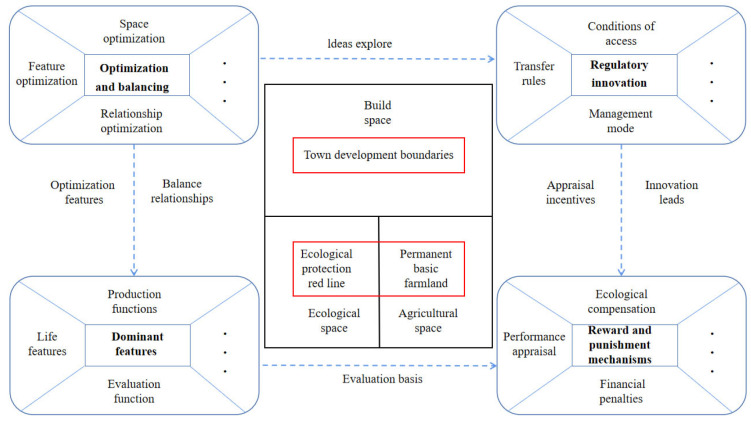
Logical framework for territorial space optimization.

Ecological security is a national strategy, and the national level is focusing more on ecological well-being such as ecological barrier restoration and resource security in the study area. Local governments should pay more attention to how to the priorities of regional development; they should enhance the first place of cities to promote employment, and protect citizens’ welfare that achieves economic and social leapfrog development. The different heights of national and local standings inevitably lead to different trade-offs on the spatial functions of the territorial space. To address the above-mentioned issues in the Aksu River Basin, we made a logical framework for territorial space optimization (Figure 13) as a key point to the trade-offs proposed from four aspects.

(1) Rationalize the relationship between development and conservation.

The unique natural conditions of the Aksu River Basin are so different that the contradiction of unbalanced and insufficient development of human-land relations has been highly prominent. How to promote the integrated development of “three types of space” on the existing location conditions and break the zero-sum game presents a dilemma which makes people think deeply [57]. The existing successful practice is to decentralize ecological space so that it plays a weak functional presence in agricultural space and urban space, but plays a strong ecological function in the overall spatial pattern. It should be especially noted that a larger the proportion of ecological space is not necessarily better. In the dynamic coordination of the “three types of space”, we must not fall into the trap of the idea of protection for the sake of protection, and must not make overly simplified decisions when things go wrong [58].

(2) Combination of flexible space and rigid bottom line.

The rigid bottom line is the blueprint for conducting spatial use control of the territorial space, which mainly has institutional rigidity, spatial rigidity, and scale rigidity [59]. The corresponding flexible space mainly has governance, functional, and structural flexibility. The rigid bottom line includes the ecological red line, permanent basic agricultural land red line, and urban development boundary red line. The “Town-Agriculture-Ecological Space” concept focuses more on rigid boundary control. The reservation of flexible space requires special policies from relevant state departments to guarantee it [60]. For the middle and lower reaches, with the relocation of heavy pollution and high energy consumption projects in the Aksu River Basin to the new local industrial parks, it is more significant to raise the level of technology and supporting facilities guarantee in the new zones (industrial zones), in addition to meeting the land supply. To solve such problems, it is necessary to rely on the reserved flexible space to find a breakthrough.

(3) Implementing precise ecological compensation.

Implementing ecological priorities can largely slow down the development of areas classified as ecologically important, and in this case, precise ecological compensation is particularly important. The model of precise ecological compensation is, in general, expressed as the redistribution of ecological compensation at the provincial and municipal levels based on the national level by setting different compensation standards, which are targeted to improve the rationality of compensation [61] and can effectively improve the motivation of local residents [62]. However, the premise is that a proven identification scheme and amount accounting must be developed [63].

(4) Inter-basin integration development.

The planning of leading industries in the Aksu River Basin is highly convergent. Currently, the advantages of the scale of industrial parks should be used uniformly to release development vitality for industry development. This can promote the formation of a new pattern of territorial space development and protection in the Aksu River Basin and the Tarim River Basin. The study area can build an urban belt along the river in the future to promote high-quality regional development [64]. Meanwhile, with the advantage of international port cities, we can also build international logistics and trade hubs with the Kyrgyz Republic and other countries through “China-Kyrgyzstan-Uzbekistan International Railway” to achieve further integration and prosperity of the transnational “Great Aksu River Basin”.

### 4.2. Practice Implication

Based on a cross-administrative watershed perspective, this study takes the Aksu River basin in southwestern Xinjiang, China, as an example, and innovatively constructs an integrated model that builds a coupled AHP-entropy weight comprehensive evaluation method, ARCGIS spatial identification analysis, coefficient of variation-TOPSIS method, and NRCA. It focuses on solving the problems of territorial space identification and optimization at the watershed scale and the elemental game relationship between different functional spaces, providing a new perspective for the study of territorial space optimization at the middle and macro levels. It also enriches the theory of sustainable development and territorial utilization in arid region watersheds, and provides experience and reference to promote the solution of similar problems.

### 4.3. Limitation and Future Directions

Due to the limited time of the study and the limited precision of the data used and personal knowledge, there is still room for optimization in the study. First, there are many more options for weight matching combinations for the combined subjective and objective weighting method used in this paper. If it is possible to compare various combinations of methods to choose the best, this will make the structure of the article more hierarchical. Second, the Aksu River Basin is relatively closed, located on the border between China and the Kyrgyz Republic, and far from developed cities. Due to the cross-administrative situation, it is influenced by the different policy objectives, development orientation, and historical basis of land spatial planning in different regions. Therefore, the positioning of the territorial and spatial functions of the Aksu River Basin should be aligned with the national macro-strategy, strengthen the links outside of region, and maintain the openness, dynamism and security of the system.

## 5. Conclusions

In this paper, we have chosen to follow the integrated approach of “characterization-suitability evaluation-conflict analysis-optimization” in the study of arid region watershed territorial space, and explored the characteristics of urban-agricultural-ecological space pattern changes, spatial conflicts, layout optimization, etc. The main conclusions are as follows.

(1) From 2000 to 2020, the greatest proportion of the territorial space in the study area has been ecological space, followed by agricultural space and then urban space. The distribution of ecological space is concentrated in the high mountain glacier snow cover area, the southern desert margin area, and the edge of irrigated agricultural area in the Aksu River Basin. Agricultural space is mainly in the irrigated agricultural area in the southeastern plain of the basin. Urban space is mainly concentrated in the administrative centers at the regional and county levels with high administrative roles.

(2) The ecological spatial conflicts are mainly distributed in different spatial transition areas of ecological vulnerability. The agricultural spatial conflicts are mainly distributed in the ecological protection red line in the downstream of the watershed. The urban spatial conflicts are mainly distributed in the plain oasis in the southeastern part of the watershed. The efficiency of territorial space utilization in the Aksu River Basin is low, and the town-agriculture-ecological space difference is obvious.

(3) After optimization, six functional areas should be designated based on the division of the territorial space of the Aksu River Basin into agricultural space, ecological space, and urban space, which are the basic rural land protection zone, rural development zone, ecological protection red line zone, ecological control zone, urban development zone, and industrial supporting construction zone.

## Figures and Tables

**Table 1:** Classification table of territorial space and land use in Aksu River Basin.

The Primary Classification	The Secondary Classification
Urban space	Urban land; Land for industrial and mining construction
Agricultural space	Land for rural settlements; Paddy land; Irrigated land
Ecological space	Forest land; Shrub land; Sparse wood; Other woodland; High coverage grassland; Moderate coverage grassland; Low coverage grassland; Canal; Lake; Reservoir; Glacier; Shoaly land; Sand land; Saline land; Marshland; Bare land; Bare rock; Gravel land

**Table 2:** Table of three types of spatial suitability evaluation index system.

The Target Layer	Index Layer	The Classification Standard (Points)
Ecological spatial suitability	1	<1600 = 4; 1600~2600 = 3; 2600~3700 = 2; >3700 = 1
	2	≥25° = 4; 25°~15° = 3; 5°~15° = 2; <5° = 1
	3	≥0.7 = 4; 0.56~0.7 = 3; 0.41~0.55 = 2 ; <0.4 = 1
	4	<5 = 4; 5~15 = 3; 15~35 = 2; >35 = 1
	5	Slight, mild = 4; Moderate and intense = 3; Very severe = 2; Extremely severe = 1
Agricultural spatial suitability	1	<1100 m = 4; 1100~1300 m = 3; 1300~1500 m = 2; >1500 m = 1
	2	<6° = 4; 6°~15° = 3; 15~25° = 2; ≥25° = 1
	6	<200 m = 4; 200~500 m = 3; 500~900 m = 2; >1000 m = 1
	7	Very mild = 4; Mild and below = 3; Moderate to intense = 2; Extremely intense = 1
	8	Arable land (including garden land) = 4; facility agricultural land = 3; Woodland and meadows =2; Other land classes = 1
	9	<1100 m = 4; 1100~3300 m = 3; 3300~5000 m = 2; >5000 m = 1
	10	<10% = 4; 10~40% = 3; 40~65% = 2; ≥65% = 1
	11	>40,000 = 4; 40,000~38,000 = 3; 38,000~36,000 = 2; ≤36,000 = 1
Urban spatial suitability	1	<1100 = 4; 1100~1300 = 3; 1300~1500 = 2; >1500 = 1
	2	<8° = 4; 8°~15° = 2; 15°~25° = 1; ≥25° = 1
	12	None occurrence area = 4; Low occurrence area = 3; Middle occurrence area = 2; High occurrence area = 1
	13	>3000 = 4; 1000~3000 = 3; 1000~500 = 2; <500 = 1
	14	>130 = 4; 90~130 = 3; 60~90 = 2; ≤60 = 1
	15	<1000 = 4; 1000~3000 = 3; 3000~6000 = 2; >6000 = 1
	16	>800 = 4; 800~500 = 3; 500~300 = 2; <300 = 1

1. Altitude; 2. Slope; 3. Vegetation coverage; 4. Relief amplitude; 5. soil erodibility; 6. Country roads; 7. Soil erosion intensity; 8. Land use status; 9. River; 10. Sand content of soil; 11. Accumulated temperature; 12. Geological disasters; 13. Population density; 14. Freshwater resource richness; 15. Distance of main road; 16. GDP.

**Table 3:** Definition of indicators.

	Indicator Name	Indicator Implication	Data Source
Urban space utilization efficiency	Construction land area per capita (m^2^/person)	Percentage of urban construction land area and urban resident population	Statistical Yearbook of Xinjiang Uygur Autonomous Region 2010 and 2020, Yearbook of Urban Construction of Xinjiang Uygur Autonomous Region 2010 and 2020, statistical bulletins of relevant counties and cities
	Land average values added by secondary and tertiary industries (CNY/person)	Percentage of the added value of urban secondary and tertiary industries to the urban construction land area	
	Regional GDP of 10,000 CNY per unit of construction land (10,000 CNY/km^2^)	Percentage of urban construction land area to regional GDP	
Agricultural space utilization efficiency	Arable land per capita (hectare/person)	Percentage of arable land area to household population	
	Gross agricultural product per capita (CNY/person)	Percentage of agricultural GDP to household population	
	Crop management area per capita (hectare/person)	Percentage of crop sown area to the household population in the current year	
	Gross agricultural product per land CNY/hectare)	Percentage of gross agricultural product to sown area	
Ecological space use efficiency	Ecological land use area (hectares)	Percentage of the land scale providing ecological service functions to the overall land scale	
	Land use area for forest, irrigation and grass (hectares)	Percentage of forest land, shrub land and grassland in the overall ecological land	

**Table 4:** Table of spatial changes in land use from 2000 to 2020 (Measurement unit: 10 km^2^).

	Urban Space				Agricultural Space				Ecological Space			
	2000		2020		2000		2020		2000		2020	
Region	Area	Proportion	Area	Proportion	Area	Proportion	Area	Proportion	Area	Proportion	Area	Proportion
Wensu	3.08	8.70%	20.16	14.10%	1313.21	22.73%	2085.91	24.23%	12,992.28	25.05%	12,196.40	24.93%
Wushi	1.05	2.96%	4.09	2.86%	552.95	9.57%	857.96	9.97%	8483.03	16.35%	8177.15	16.71%
Awati	3.02	8.52%	9.78	6.84%	1239.18	21.45%	1675.05	19.46%	11,776.42	22.70%	11,333.78	23.17%
Ahqi	0.83	2.35%	1.21	0.85%	130.58	2.26%	166.35	1.93%	11,290.75	21.77%	11,249.82	22.99%
Alaer	1.71	4.81%	19.12	13.37%	1189.89	20.59%	1980.48	23.01%	2735.44	5.27%	1927.43	3.94%
Aksu	25.74	72.66%	88.62	61.98%	1352.03	23.40%	1843.02	21.41%	4594.17	8.86%	4040.28	8.26%
Total	35.42	100%	142.98	100%	5777.84	100%	8608.77	24.23%	51,872.09	100%	48,924.85	100%
Space occupancy ratio	0.06%		0.25%		10.02%		14.93%		89.94%		84.83%	

**Table 5:** Dynamic table of land use change from 2000 to 2020 (Measurement unit: km^2^).

		Urban Space	Agricultural Space	Ecological Space
**Single land use**	**Urban space**	26.17	62.59	68.84
	**Agricultural space**	3.26	4327.08	4463.41
	**Ecological space**	1.32	553.68	48,156.45
**Comprehensive land use**		13.75	2.60	−0.25

**Table 6:** Table of spatial types of urban towns in the Aksu River Basin (proportion: %, area: km^2^).

Regions	Most Suitable Area		Suitable Area		Less Suitable Area		Unsuitable Area	
	Area	Proportion	Area	Proportion	Area	Proportion	Area	Proportion
Wensu	317.87	4.50%	5605.80	36.17%	4087.33	18.81%	4106.11	33.02%
Wushi	972.80	13.77%	5166.81	33.34%	1788.71	8.23%	982.47	7.90%
Awati	76.91	1.09%	0.00	0.00%	7205.69	33.17%	5569.23	44.79%
Alaer	2094.77	29.65%	0.00	0.00%	1556.96	7.17%	194.46	1.56%
Aksu	3550.03	50.25%	1035.14	6.68%	3547.10	16.33%	1278.88	10.29%
Ahqi	52.02	0.74%	3691.89	23.82%	3538.16	16.29%	302.73	2.43%
Total	7064.41	100%	15,499.63	100%	21,723.96	100%	12,433.88	100%

**Table 7:** Table of agricultural spatial status in the Aksu River Basin (proportion: %, area: km^2^).

Regions	Most Suitable Area		Suitable Area		Less Suitable Area		Unsuitable Area	
	Area	Proportion	Area	Proportion	Area	Proportion	Area	Proportion
Wensu	256.19	26.82%	485.67	19.31%	167.29	17.80%	485.67	26.49%
Wushi	335.67	35.14%	230.95	9.18%	243.42	25.90%	230.95	18.66%
Awati	164.24	17.19%	766.67	30.48%	88.94	9.46%	766.67	4.19%
Alaer	58.32	6.11%	413.73	16.45%	49.44	5.26%	413.73	1.68%
Aksu	133.52	13.98%	601.57	23.92%	209.23	22.26%	601.57	14.20%
Ahqi	7.20	0.75%	16.80	0.67%	181.65	19.32%	16.80	34.78%
Total	955.14	100%	2515.39	100%	939.97	100.00%	2515.39	100%

**Table 8:** Table of ecological spatial suitability structure of Aksu River Basin (percentage: %, area: km^2^).

Regions	Most Suitable Area		Suitable Area		Less Suitable Area		Unsuitable Area	
	Area	Proportion	Area	Proportion	Area	Proportion	Area	Proportion
Wensu	1571.84	26.27%	4922.89	30.35%	3615.35	27.62%	4062.03	18.72%
Wushi	616.75	10.31%	2797.48	17.25%	2249.42	17.18%	3249.97	14.98%
Awati	661.62	11.06%	1115.78	6.88%	1464.24	11.19%	9717.41	44.79%
Alaer	1361.56	22.75%	685.61	4.23%	755.21	5.77%	1113.77	5.13%
Aksu	775.26	12.96%	1015.78	6.26%	2267.86	17.32%	1849.80	8.53%
Ahqi	997.15	16.66%	5683.98	35.04%	2738.07	20.92%	1703.96	7.85%
Total	5984.17	100%	16,221.52	100%	13,090.00	100%	21,696.00	100%

**Table 9:** Table of comparative advantage index of territorial spatial functions in Aksu River Basin.

Regions	Spatial Advantage Index of Urban Space	Spatial Advantage Index of Agricultural Space	Spatial Advantage Index of Ecological Space	Advantageous Function Space
Wensu	0.00035100	0.02733934	0.15780211	Ecological space
Wushi	0.00013024	0.01229570	0.11908949	Ecological space
Awati	0.00017663	0.02317497	0.15053145	Ecological space
Alaer	0.00001544	0.00200601	0.15601655	Ecological space
Aksu	0.00023166	0.03258494	0.03030857	Agricultural space
Ahqi	0.00143575	0.02990507	0.06101067	Ecological space

**Table 10:** Territorial space optimization zoning structure of Aksu River Basin.

Space Types	Functional Zoning	Area	Proportion	Space Area	Spatial Proportion
Ecological space	Ecological red line zone	24,819.65 km^2^	43.03%	48,729.98 km^2^	84.48%
	Ecological control zone	23,910.33 km^2^	41.45%		
Agricultural space	Basic farmland protection zone	8496.90 km^2^	14.73%	8793.87 km^2^	15.25%
	Rural development area	296.97 km^2^	0.51%		
Urban space	Urban development area	111.78 km^2^	0.19%	157.61 km^2^	0.27%
	Industrial support construction area	45.83 km^2^	0.08%		

**Table 11:** Territorial space optimization zoning structure of Aksu River Basin (Area: km^2^).

	2020 Urban Space		2030 Urban Space		2020 Agricultural Space		2030 Agricultural Space		2020 Ecological Space		2030 Ecological Space	
	Area	Proportion	Area	Proportion	Area	Proportion	Area	Proportion	Area	Proportion	Area	Proportion
Wensu	20.16	14.10%	27.06	17.17%	2085.91	24.23%	2107.94	23.97%	12,196.40	24.93%	12,166.97	24.97%
Wushi	4.09	2.86%	8.95	5.68%	857.96	9.97%	845.09	9.61%	8177.15	16.71%	8185.09	16.80%
Awati	9.78	6.84%	13.22	8.39%	1675.05	19.46%	1735.11	19.73%	11,333.78	23.17%	11,270.28	23.13%
Alaer	1.21	0.85%	1.12	0.71%	166.35	1.93%	144.91	1.65%	11,249.82	22.99%	11,270.53	23.13%
Aksu	19.12	13.37%	14.41	9.14%	1980.48	23.01%	2027.12	23.05%	1927.43	3.94%	1885.51	3.87%
Ahqi	88.62	61.98%	92.84	58.91%	1843.02	21.41%	1933.82	21.99%	4040.28	8.26%	3945.27	8.10%
Total	142.98	100%	157.6	100%	8608.77	100.00%	8793.99	100.00%	48924.85	100.00%	48723.65	100.00%

## Data Availability

Not applicable.
